# Tailoring Polyetherimide Ultrafiltration Membranes via Non-Solvent- and Vapor-Induced Phase Separation: Effects of Fabrication Pathway and Membrane Formulation on Morphology and Bovine Serum Albumin Separation Performance

**DOI:** 10.3390/membranes16090288

**Published:** 2026-08-28

**Authors:** Mohammad Hosein Moghadasin, Masoud Salavati, Mohammed Majdoub, Ahmed Al-Ostaz, Alexander M. Lopez, Sasan Nouranian

**Affiliations:** 1 Department of Chemical Engineering, University of Mississippi, Oxford, MS 38677, USA; 2 Center for Graphene Research and Innovation, University of Mississippi, Oxford, MS 38677, USA; 3 Department of Civil Engineering, University of Mississippi, Oxford, MS 38677, USA; 4 Department of Mathematics and Physical Sciences, Rogers State University, Claremore, OK 74017, USA

**Keywords:** polyetherimide, graphene nanoplatelets, membrane, nonsolvent-induced phase separation, vapor-induced phase separation

## Abstract

Polyetherimide (PEI) ultrafiltration membranes were fabricated for bovine serum albumin (BSA) separation using nonsolvent-induced phase separation (NIPS) and vapor-induced phase separation (VIPS). In the NIPS approach, membranes containing varying amounts of Pluronic P-123, with or without graphene nanoplatelets (GNPs), were prepared. Pluronic P-123 acted as a pore-forming agent, while GNPs enhanced permeability without compromising rejection. The membrane with 1 wt.% Pluronic P-123 achieved a pure water flux (PWF) of 355 LMH and 90% BSA rejection. Incorporating 1 wt.% GNPs further improved performance to 747 LMH and 98% rejection. VIPS membranes were fabricated using the same composition as N10, containing 17 wt.% PEI and 1 wt.% Pluronic P-123 without GNPs, to isolate the effect of the fabrication pathway. Increasing vapor exposure time reduced PWF but generally improved BSA rejection, whereas higher RH increased PWF while decreasing rejection. The composition-matched NIPS membrane, N10, exhibited a PWF of 355 LMH and approximately 91% BSA rejection. In comparison, the highest PWF and BSA rejection among the VIPS membranes were 91 LMH and 76.5%, respectively, and the selected balanced VIPS condition at 30 min and 65% RH yielded approximately 49 LMH and 55% rejection. The higher performance of N11, which achieved 747 LMH and 98% rejection, is attributed to GNP incorporation within the NIPS series and was not used to isolate the fabrication-pathway effect. Overall, the composition-matched comparison demonstrates that NIPS produced a more favorable morphology and BSA separation performance than VIPS under the investigated conditions.

## 1. Introduction

Membrane-based separation systems are widely regarded as more sustainable than traditional separation processes with applications spanning desalination, water and wastewater treatment, industrial effluents, water reclamation, energy storage, and carbon dioxide capture [1,2,3,4,5,6,7,8,9,10,11,12,13,14]. Membrane performance is often defined by the thickness and resultant morphology of its critical separation or active layer. Considerable research has been devoted to the development of precise and scalable fabrication strategies for both polymeric and inorganic membrane systems, including phase inversion, interfacial polymerization, electrospinning, sol–gel synthesis, and ceramic processing techniques [15,16,17,18,19,20].

Although NIPS and VIPS have been investigated across several polymer systems, direct composition-matched comparisons linking the fabrication pathway to both membrane morphology and application-specific separation performance remain comparatively limited. Nonsolvent-induced phase separation (NIPS) is the most common method for producing asymmetric polymeric membranes [21,22,23]. NIPS involves a casting solution, where a polymer and a filler (e.g., nanomaterial, porogen) are dissolved or dispersed within a compatible solvent. This solution is then shaped into the desired form factor (e.g., flat sheet or hollow fiber) and immersed in a nonsolvent. The latter is miscible with the initial solvent but acts as a poor solvent for the polymer. The resultant mass transfer induces phase inversion of the membrane material. Moreover, the rapid exchange of solvent and nonsolvent in NIPS typically induces phase inversion into an asymmetric structure—a polymer-rich dense skin layer forms at the interface (solution/nonsolvent bath), supported by a more porous substructure beneath the skin layer [21].

In contrast with NIPS, vapor-induced phase separation (VIPS) is a casting technique that relies on a nonsolvent vapor, usually water vapor, penetrating the casting solution to induce the phase inversion process. VIPS can be considered a variant of NIPS with slower mass transfer rates and more adjustable phase inversion parameters, due to the additional diffusion step required during VIPS [24,25]. The slower formation of the polymer matrix leads to the appearance of ultra-small pores; however, a variety of pore sizes and membrane morphologies can be achieved through effective control of the VIPS parameters [26]. Specifically, there are four main morphologies commonly achieved during VIPS casting: (1) symmetric cellular, (2) asymmetric cellular, (3) symmetric nodular, and (4) sponge-like bi-continuous. Membrane casting parameters and casting solution formulations both contribute to morphology formation. This versatility makes VIPS an attractive option in designing membrane materials; however, the added complexity of controlling humidity and vapor exposure within VIPS results in added synthesis complexity. NIPS involves rapid exchange of solvent and nonsolvent, typically producing an asymmetric membrane. This fast, out-of-equilibrium demixing often yields finger-like macrovoid pores in the cross-section of the membrane, if conditions allow, or a sponge-like structure if macrovoid formation is suppressed [27]. In contrast, VIPS uses a nonsolvent vapor to induce phase separation more gradually. The slower vapor diffusion means the polymer film absorbs nonsolvent over a controlled period, leading to a more uniform precipitation. As a result, VIPS can produce a highly porous surface (due to the lower polymer concentration at the air interface during casting) and can be tuned to generate various pore morphologies, from cellular structures to interconnected bi-continuous networks by adjusting the humidity and exposure time. This tunability makes VIPS a powerful method for tailoring membrane structure to specific applications [28].

Currently, researchers have explored various applications for VIPS membranes with the majority focused on microfiltration (MF) and ultrafiltration (UF) [25]. In one study, Dehban et al. [29] fabricated polyphenylsulfone (PPSU)/polyether sulfone (PES)/nanosilica (SiO_2_) composite UF membranes with a unique sponge-like, bi-continuous morphology through a combination of VIPS and NIPS. Their study explored various parameters such as VIPS time, PPSU:PES blending ratio, polymer concentration, and SiO_2_ nanoparticles concentration to optimize the membrane performance. Their results revealed the critical role of VIPS in influencing the membrane structure. In another work, Marino et al. [30] fabricated poly(vinylidene fluoride) (PVDF) membranes for MF and UF applications via the modulation of polyethylene glycol (PEG) concentrations within the casting solutions, while employing NIPS and VIPS. Their results demonstrated how adjusting the PEG content and vapor exposure time produced membranes with varying pore diameters and bi-continuous structures. In membranes produced through NIPS, macrovoids and spongy structures were observed, with their formation influenced by PEG concentration. When VIPS was used, the resulting membranes displayed a symmetric, bi-continuous morphology. A study by Menut et al. [31] demonstrated the production of asymmetric cellular morphologies within polyetherimide (PEI) membranes prepared using VIPS. This morphology was achieved through a solvent gradient present during the casting of the membrane, resulting in a decreasing cellular size from the top to the bottom layer. An asymmetric finger-like morphology can be achieved via fast phase separation processes, typically through short vapor exposure times within VIPS. This limited exposure prevents complete phase inversion across the polymer solution. As a result, the bottom layers typically undergo the NIPS method with a nonsolvent coagulation bath, emphasizing the use of both VIPS and NIPS casting methods to develop this unique morphology.

The previously described research highlights the flexibility of the VIPS process but does not fully explore its potential for task-specific membrane performance based on VIPS-derived membrane morphologies. Further investigation is needed to compare membranes produced by VIPS and NIPS methods for various applications, particularly those that link membrane morphology to performance indicators such as water flux and solute rejection. Previous studies have demonstrated the influence of NIPS and VIPS processing conditions on membrane morphology in polymer systems including PPSU/PES, PVDF, and PEI; however, these investigations have primarily focused on morphology development and process optimization. The present study extends these findings by using a composition-matched PEI formulation to directly compare NIPS and VIPS in terms of both membrane morphology and BSA separation performance, while separately evaluating the effects of Pluronic P-123 and GNP incorporation within the NIPS series. This study seeks to address this gap by comparing the NIPS and VIPS methods to evaluate their performance in separating bovine serum albumin (BSA) from water using PEI UF membranes. It also demonstrates the role of porogens and nanoadditives in water treatment membranes by incorporating Pluronic P-123 (a triblock copolymer, i.e., poly(ethylene oxide)-b-poly(propylene oxide)-b-poly(ethylene oxide) or PEO-*b*-PPO-*b*-PEO) and graphene nanoplatelets (GNPs) into the membrane casting solution. PEI was selected because of its favorable thermal, chemical, and mechanical stability, which can support membrane durability. NMP was used because of its strong solvating ability for PEI; however, its health and environmental concerns highlight the importance of solvent recovery and the future evaluation of safer solvent alternatives. In the NIPS method, membranes were made with varying amounts of Pluronic P-123 in the presence or absence of GNPs. For the VIPS method, membranes were made without GNPs to assess the impact of vapor exposure time and relative humidity on membrane morphology. To isolate the effect of the fabrication pathway, the VIPS membranes were compared specifically with N10, the composition-matched NIPS membrane containing 1 wt.% Pluronic P-123 and no GNPs; the GNP-containing NIPS membranes were evaluated separately to determine the effect of the nanoadditive.

## 2. Materials and Methods

### 2.1. Materials

PEI (melt index: 18 g/10 min) and N-Methyl-2-pyrrolidone (NMP) (anhydrous, 99.5%) were purchased from MilliporeSigma (St. Louis, MO, USA). Pluronic P-123 (average Mn: ~5800) was also purchased from MilliporeSigma. Pluronic P-123 is a triblock copolymer PEO-*b*-PPO-*b*-PEO and acts as a porogen in the PEI UF membranes due to its unique amphiphilic structure, enabling the formation of well-defined pores [32]. As a result, membranes with high porosity and enhanced performance are obtained. BSA (molecular weight: 66,000 g/mol) was purchased from MilliporeSigma and selected as a representative model protein for evaluating the separation and fouling behavior of the PEI UF membranes. Accordingly, the separation results reported herein are specific to BSA under the investigated filtration conditions and should not be generalized to other macromolecular solutes or mixed-contaminant feeds without additional evaluation. Phosphate-buffered saline (PBS) was purchased from Thermo Fisher Scientific (Waltham, MA, USA) and used to maintain the pH of the BSA solution at 7.4 ± 0.1. GNPs (grade: xGnP C-750) were purchased from XG Sciences, Inc. (Lansing, MI, USA).

### 2.2. Membrane Fabrication

**Figure 1:**
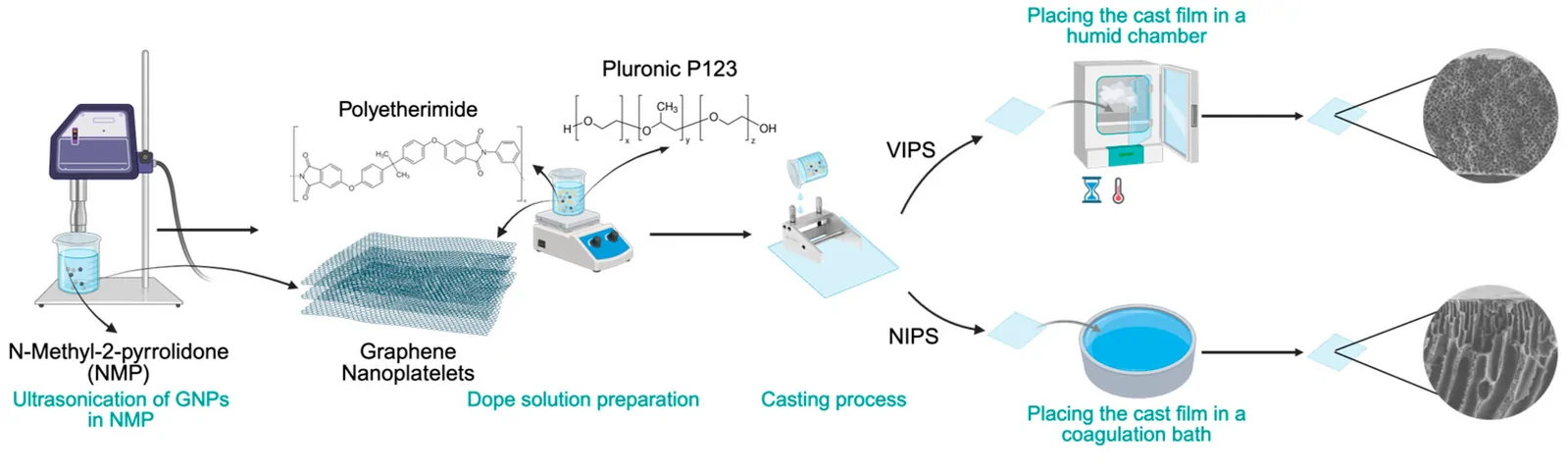
Schematics of membrane fabrication. Created in BioRender. Moghadasin, M. (2026). https://BioRender.com/qdilujc.

The UF membranes in this work were fabricated using the NIPS and VIPS methods. PEI was used as the base polymer, NMP as the solvent, Pluronic P-123 as the porogen, and GNPs as a nanoadditive. The main steps of the membrane fabrication process are depicted in Figure 1. First, the PEI granules were dried in an oven at 60 °C for 24 h. To prepare the casting solutions, GNPs were first added to NMP to make a 20 mL solution based on the compositions provided in Table 1. GNPs were dispersed in the solvent by ultrasonication using a Qsonica Q700 probe sonicator (Newtown, CT, USA) (10 s ON/5 s OFF; 20 kHz; 50% power) for 15 min. Next, Pluronic P-123 and PEI were added to the mixture to achieve the desired composition (Table 1). The mixture was continuously stirred for 24 h at 65 °C to form a homogeneous solution. Next, the solution was stored in a desiccator at room temperature for 24 h to degas. The solution was then cast on a glass plate using a micrometer adjustable film applicator (model EQ-Se-KTQ-100, MTI Corporation, Richmond, CA, USA) with a thickness of 100 µm. For samples made with the NIPS method, the plate was placed in a water bath at room temperature for 24 h to finish the solvent/nonsolvent exchange and eliminate any leftover solvent. The films were transferred directly to the coagulation bath without an intentional vapor-exposure period; therefore, ambient laboratory relative humidity was not controlled as an experimental parameter during NIPS fabrication. For samples made with the VIPS method, the plate was placed in an oven at 50 °C with a target RH and vapor exposure time (Table 2). Next, the plate was taken out of the oven and placed in a water bath at room temperature for 24 h to complete the solvent/nonsolvent exchange and remove any remaining solvent.

For all samples fabricated using the VIPS method, the Pluronic P-123 content was maintained at 1 wt.%, and GNPs were excluded from the formulations. Accordingly, N10, containing 17 wt.% PEI, 1 wt.% Pluronic P-123, and no GNPs, served as the composition-matched NIPS reference for all V10-x membranes. N00 served as the neat PEI control. Comparisons between N10 and the V10-x series were therefore used to isolate the effect of the phase-separation pathway, whereas comparisons involving N11 and N21 were used only to evaluate the effect of GNP incorporation within the NIPS series. The VIPS membranes, V10-1 to V10-9, were fabricated by varying RH and vapor exposure time, as presented in Table 2.

### 2.3. Membrane Characterization and Performance Testing Methods

The membrane surface hydrophilicity was studied by measuring the water contact angle (WCA) at room temperature using an Attension optical tensiometer (Biolin Scientific, Gothenburg, Sweden), incorporating a single-liquid automatic dispenser, an inbuilt NAVITAR (Model 520931), and OneAttension software (version 2.4). To reduce experimental errors, an average of five measurements were made and are reported herein. Additionally, attenuated total reflectance-Fourier transform infrared spectroscopy (ATR-FTIR) was used to determine the presence of functional groups on the membrane surfaces and to study their chemical compositions using a Nicolet iS50 instrument (Thermo Fisher Scientific, Madison, WI, USA) in transmission mode using the ATR sampling technique. All FTIR spectra were recorded in the range of 500–4000 cm^−1^ at a resolution of 8 cm^−1^ with an accumulation of 32 scans.

The overall porosities (ε) of the fabricated membranes were determined using the gravimetric method [33]:

(1) $$\varepsilon =\frac{m_{w}-m_{d}}{AL\rho_{w}},$$

where $m_{w}$ (kg) represents the wet membrane weight, $m_{d}$ (kg) is the dried membrane weight, L (m) is the membrane thickness, A (m^2^) is the membrane effective area, and $\rho_{w}$ (997 kg/m^3^) is the density of pure water at 25 °C. Furthermore, the apparent mean hydraulic pore radii ($r_{m}$) of the fabricated membranes were estimated using the Guerout–Elford–Ferry equation [33,34]:

(2) $$r_{m}=\sqrt{\frac{8\mu LQ(2.9-1.75\varepsilon )}{\varepsilon A∆P}},$$

where μ (8.9 × 10^−4^ Pa s) is the viscosity of water at 25 °C, ΔP (Pa) is the transmembrane pressure, and Q (m^3^ s^−1^) is the volumetric flow rate of pure water. It should be noted that the pore size calculated using this equation represents an apparent hydraulic pore size estimated from water permeation behavior, rather than a direct measurement of the selective skin-layer pore size. Therefore, these values should be interpreted as effective transport-related pore sizes for comparing membrane samples, not as the actual pore-throat size governing solute rejection. As an experimental limitation, nominal membrane thickness was used to estimate flux as the cast solutions consistently produced membranes with an approximate 100 µm thickness and negligible variation. Because individual membrane thickness was not directly used to calculations, variations in actual thickness could introduce minor uncertainty into the calculated values.

The cross-sections and surface images of the fabricated membranes were studied using a Field Emission scanning electron microscope (Model 6500F, JEOL, Tokyo, Japan) at an accelerating voltage of 5–20 kV. To prepare samples for cross-sectional imaging, the membranes were fractured after dipping them in liquid nitrogen, whereafter they were coated with a thin layer of gold. The quasistatic tensile properties of the membranes were evaluated using a universal testing system (Model 5982, Instron Corp., Norwood, MA, USA) equipped with a 5 kN load cell, a crosshead speed of 10 mm/min, and a video extensometer (Model 2663-902, Instron Corp., Norwood, MA, USA) for accurate strain measurement, based on the ASTM D-882 standard [35]. To ensure proper statistics, five specimens were tested per membrane composition (Table 1 and Table 2).

The pure water fluxes (PWFs) of the fabricated membranes were measured using a high-pressure, stirred cell (dead-end filtration) kit (Model HP4750, Sterlitech, Corp., Auburn, WA, USA) with an effective area of 8.3 cm^2^. Initially, all membranes were compacted with deionized (DI) water for 30 min to reach a stable flux. Subsequently, the flux was recorded every 5 min over a period of 45 min under an operating pressure of 0.6 MPa according to the preliminary experiments. The PWF (J) was calculated using the following equation [33]:

(3) $$J=\frac{V}{A∆t},$$

where J represents the PWF in units of L m^−2^ h^−1^ (LMH), A is the effective membrane area in square meters (m^2^), V is the permeate volume in liters (L), and ∆t is the operating time (h). After recording the PWF, pure water was replaced with a 100 ppm BSA solution with its pH maintained at 7.4 ± 0.1 using PBS, and the membrane rejection was assessed using this solution. The BSA rejection (R) was calculated using the following equation [33]:

(4) $$R=(1-\frac{C_{p}}{C_{f}})\times 100,$$

where $C_{p}$ represents the permeate solution concentration and $C_{f}$ is the feed solution concentration (100 ppm). The BSA concentration was determined using a UV-vis spectrophotometer at 595 nm similar to the method used by Pishbin et al. [33]. All filtration experiments were conducted at room temperature. For each membrane condition, three membrane specimens (n=3) were tested, and the results are presented as the mean ± standard deviation. The specimens comprised samples obtained from separate cast sheets and samples cut from spatially distinct regions of larger cast sheets. For the VIPS series, the effects of relative humidity (40%, 65%, and 90%), vapor exposure time (15, 30, and 60 min), and their interaction on PWF and BSA rejection were evaluated using separate two-way analyses of variance (ANOVA). The N10 membrane, represented by zero exposure time, was excluded from the ANOVA because it was fabricated using NIPS and was not part of the 3×3 VIPS factorial design. Statistical significance was defined at p<0.05.

Three flux measurements were conducted to investigate the membrane antifouling properties. First, the initial PWF (*J_w_*_1_) was measured, as discussed earlier. After this step, the BSA solution was replaced with DI water, and the BSA solution flux (*J_P_*) was recorded by measuring the permeate mass every 5 min for the duration of 45 min. Next, the membrane was immersed in water for 15 min and gently washed. Then, the second PWF (*J_w_*_2_) was measured for 45 min. Based on the collected data, the flux recovery ratio (*FRR*), total fouling ratio (*R_t_*), irreversible fouling ratio (*R_ir_*), and reversible fouling ratio (*R_r_*) were calculated using the following equations [33]:

(5) $$FRR=(\frac{J_{w2}}{J_{w1}})\times 100,$$

(6) $$R_{t}=(\frac{J_{w1}-J_{P}}{J_{w1}})\times 100,$$

(7) $$R_{ir}=(\frac{J_{w1}-J_{w2}}{J_{w1}})\times 100,$$

(8) $$R_{r}=(\frac{J_{w2}-J_{P}}{J_{w1}})\times 100.$$

## 3. Results and Discussion

### 3.1. Membrane Characterization

#### 3.1.1. Membrane Surface Hydrophilicity

**Figure 2:**
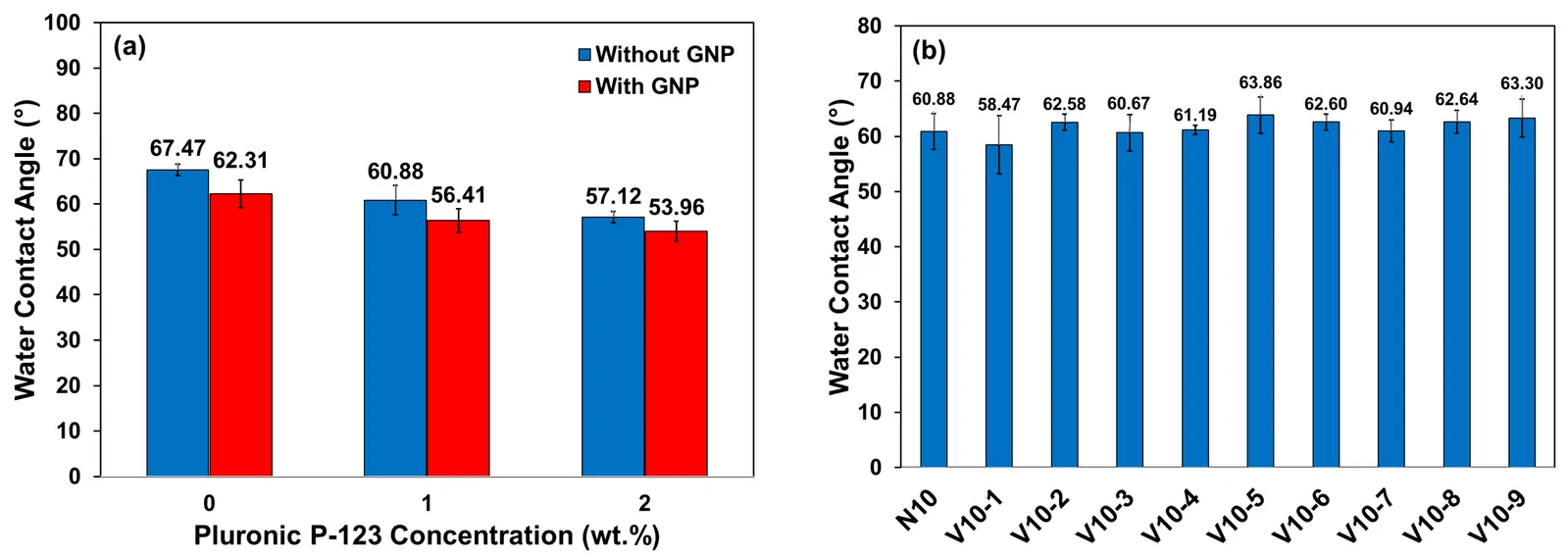
Water contact angles of the PEI membranes fabricated using (**a**) NIPS and (**b**) VIPS. Data are presented as mean ± standard deviation (*n* = 5).

In Figure 2, water contact angle (WCA) measurements of the fabricated membranes are shown. The average WCA values for the NIPS membranes generally decrease with an increase in both Pluronic P-123 and GNP contents (Figure 2a). Specifically, the WCA values drop from ~67° for N00 (no Pluronic P-123 or GNPs) to ~62° for N01 (no Pluronic P-123; 1 wt.% GNPs) and to ~61° for N10 (1 wt.% Pluronic P-123; no GNPs). This trend indicates that adding GNPs and/or Pluronic P-123 increases the hydrophilic nature of the PEI membrane surfaces. Additionally, the WCA of N10 is compared to that of the VIPS membranes, since all VIPS membranes have the same amount of Pluronic P-123 as that of N10 (i.e., 1 wt.%) without GNPs. In terms of the components, all VIPS samples are similar to N10, differing only in processing conditions. In Figure 2b, a slight increase in the WCAs with increasing vapor exposure time is observed, which suggests increased hydrophobicity and dense structure, aligning with the observed membrane permeability performance discussed later.

#### 3.1.2. Membrane Surface Functional Groups

**Figure 3:**
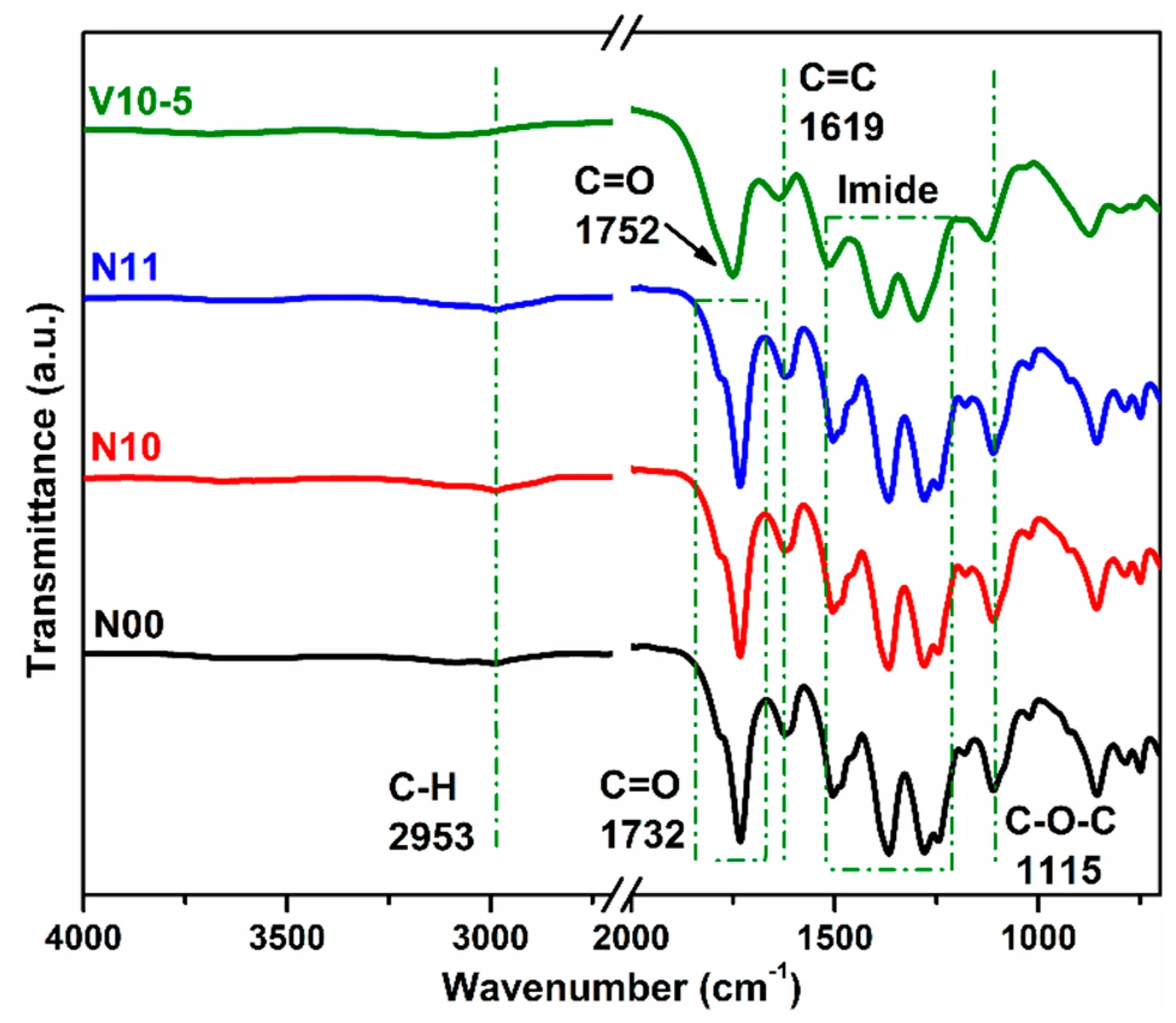
FTIR spectra of the fabricated PEI membranes.

The ATR-FTIR transmittance spectra of the membranes (Figure 3) reveal the key functional groups of the PEI membrane surface. Major peaks include the out-of-plane bending vibrations of the aromatic C-H bonds at 862 cm^−1^ [36], the stretching vibration of the C-O-C (ether) groups at 1115 cm^−1^ [37], and the asymmetric and symmetric stretching vibrations of the C-N bonds in the imide ring at 1275 cm^−1^ and 1364 cm^−1^, respectively [38,39]. Additionally, the bands at 1502 cm^−1^ and 1619 cm^−1^ correspond to the stretching vibrations of the aromatic C=C bonds in the benzene rings [40,41], while the absorption observed at 1732–1752 cm^−1^ region indicates the presence of C=O (carbonyl) group stretching vibrations [38,39]. The band at 2953 cm^−1^ represents the asymmetric stretching vibrations of the C-H bonds in methyl (CH_3_) or methylene (CH_2_) groups within the polymer backbone or side chains [39]. These peaks confirm the chemical structure of PEI and its functional groups in the membranes, consistent across all samples. The results completely align with the observations of other researchers.

A slight change in the relative band intensity and band shape of the ATR-FTIR spectrum of N11 is observed, particularly in the region corresponding to aromatic C=C bonds. These changes may be associated with the incorporation of graphitic carbon from the GNPs and changes in the local chemical environment of PEI in the presence of the graphene-based filler [42]. Interfacial interactions between graphene-based materials and PEI have been reported previously; however, the present FTIR results alone are insufficient to establish a specific PEI–GNP bonding mechanism [42]. For the V10-5 sample, a slight shift to higher wavenumbers is particularly evident for the carbonyl region, where the C=O band appears around 1752 cm^−1^ compared with the lower-wavenumber carbonyl band observed for the NIPS samples. A decrease in the relative band intensity is also observed in some regions of the V10-5 spectrum. These changes can be attributed to alterations in the polymer chain packing density and local molecular environment induced by the VIPS method, which affect the vibrational energy levels of the polymer bonds, leading to peak shifts. Changes in chain packing, intermolecular interactions, and phase-separation-induced morphology may produce such band shifts and broader or less intense absorption features, thereby influencing the FTIR response [43].

#### 3.1.3. Membrane Porosity and Apparent Mean Hydraulic Pore Radius Estimation

Table 3 and Table 4 present the porosity and apparent mean hydraulic pore sizes of the NIPS and VIPS membranes, respectively. For membranes made using the NIPS method, adding Pluronic P-123 as a porogen expectedly increases both the porosity and apparent mean hydraulic pore size. Specifically, for the N00 membrane, the porosity is about 32% and the apparent mean hydraulic pore sizes is about 26 nm (Table 3). For N10 and N20, these values rise to 35% and 38% for porosity, and 28 nm and 44 nm for apparent mean hydraulic pore size, respectively. In general, porosity and pore size are functions of the diffusion rate during the phase inversion process [44]. Adding GNPs or switching the fabrication method from NIPS to VIPS significantly affects the diffusion rate and, consequently, the morphologies, porosities, and apparent mean hydraulic pore sizes of the membranes. Comparing N00 to N01, N10 to N11, and N20 to N21 (Table 3) shows that including GNPs in the membrane composition increases the porosity and apparent mean hydraulic pore size of the membrane. In general, adding GNPs to the dope solution not only increases its viscosity but also accelerates the solvent–nonsolvent exchange rate during phase inversion due to the abundance of hydrophilic functional groups in the GNPs [44,45,46]. In the present membranes, the observed increases in porosity and apparent mean hydraulic pore size after GNP incorporation suggest that the effect of enhanced solvent–nonsolvent exchange associated with the hydrophilic GNP surface was more pronounced than the opposing effect of increased dope-solution viscosity.

Although the apparent mean hydraulic pore radii of the NIPS membranes are larger than the approximate hydrodynamic radius of BSA, these quantities describe different structural features and should not be compared directly. The Guerout–Elford–Ferry values represent effective hydraulic radii influenced by transport through the entire membrane thickness, including the highly porous finger-like substructure and macrovoids. In contrast, BSA rejection is controlled primarily by the narrowest connected pore constrictions and the integrity of the dense selective surface layer. Therefore, the porous NIPS substructure can support high water flux and produce a relatively large apparent hydraulic radius, while the considerably tighter selective skin maintains high BSA rejection.

In VIPS membranes, we determined the impact of vapor exposure time and RH on porosity and pore size (Table 4). Comparing results over short to moderate vapor exposure times (15–30 min) reveals a consistent trend, where increasing vapor exposure time leads to a decrease in porosity and an increase in apparent mean hydraulic pore sizes across all tested RH levels (40, 65, and 90%). This indicates that longer vapor exposure times promote the formation of larger pores and denser membrane structures. This can be attributed to the slower mass transfer rates in VIPS compared to NIPS, which results in limited skin layer formation, thus promoting the formation of larger and more numerous pores. Our observation herein is consistent with those of Xu et al. [47], where increasing vapor exposure time initially boosts pore size and water flux due to increased water vapor concentration and phase separation dynamics. At longer vapor exposure times (60 min), porosity and pore size continue to increase, albeit to a lesser extent. This behavior suggests the development of a more interconnected, porous structure within the membrane, as also observed in studies by Xu et al. [47] and Venault et al. [48]. Moreover, higher RH levels expedite phase separation by facilitating greater water vapor absorption into the polymer solution. This accelerates pore formation, resulting in higher porosity and larger pore sizes in the membranes. In general, both vapor exposure time and RH are found herein to be critical factors in determining membrane morphology. Longer vapor exposure times tend to decrease porosity while increasing pore size, reflecting more extensive phase separation and densification. Higher RH levels initially increase porosity and pore size due to enhanced water vapor diffusion, but these effects become more complex with extended vapor exposure time.

Conversely, the smaller apparent hydraulic radii calculated for the VIPS membranes do not necessarily indicate smaller selective-surface pores. Their lower water flux reflects the combined effects of lower bulk porosity, compact regions, increased tortuosity, and transport resistance across the entire membrane thickness. The SEM images indicate that the VIPS membranes lack the pronounced and continuous dense selective skin observed in the NIPS membranes, and their cellular pores extend closer to the top surface. Consequently, relatively open or heterogeneous connected pathways can permit BSA transport even when the whole-membrane hydraulic radius calculated from water permeation is smaller. BSA transport is therefore governed primarily by size exclusion at the narrowest connected constrictions, while the pore-size distribution, selective-layer continuity, pore connectivity, tortuosity, and protein adsorption or fouling also affect the measured rejection.

#### 3.1.4. Membrane Morphology

**Figure 4:**
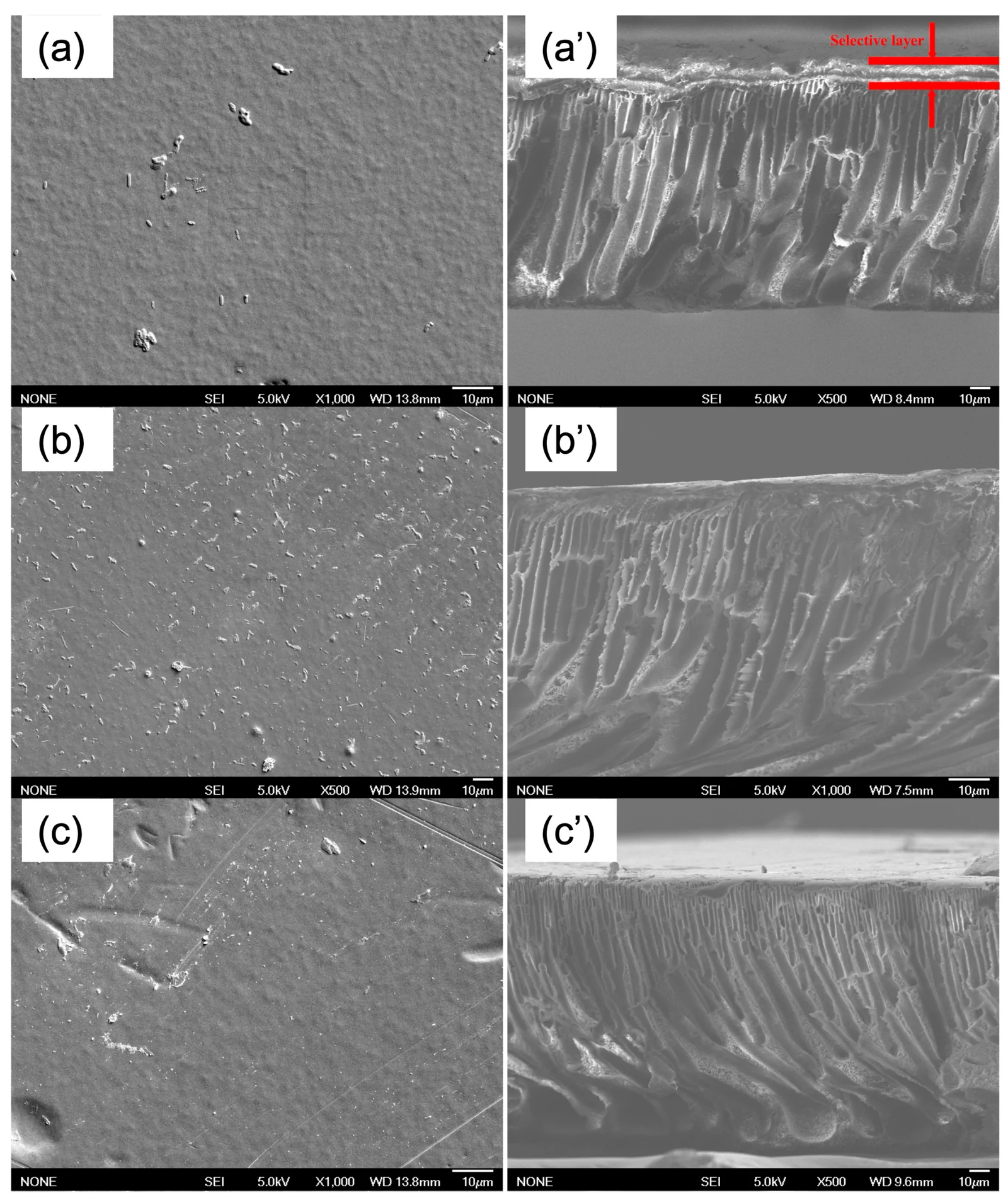

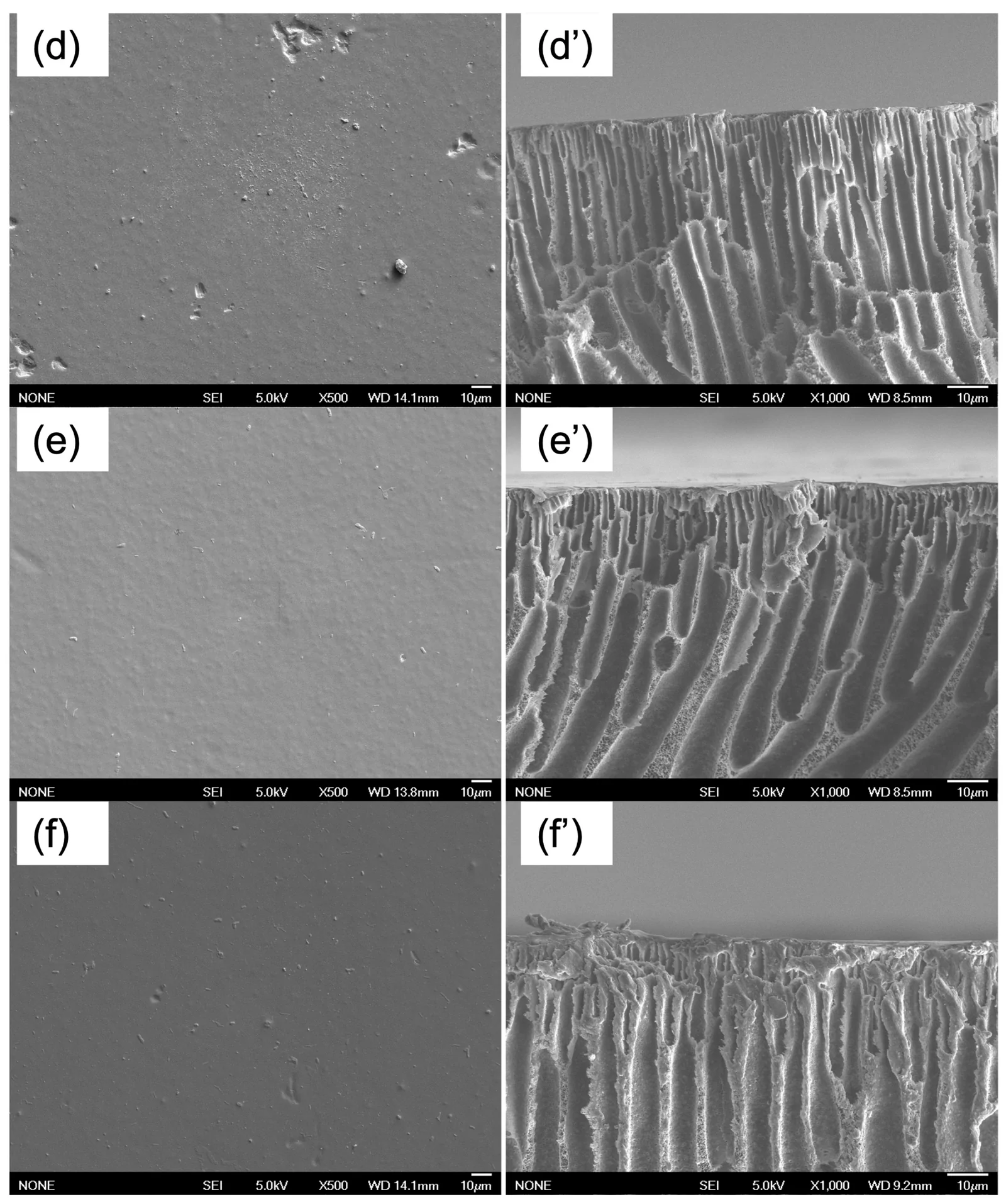
SEM images of the surfaces and cross-sections of the NIPS membranes: (**a**,**a′**): N00, (**b**,**b′**): N01, (**c**,**c′**): N10, (**d**,**d′**): N11, (**e**,**e′**): N20, (**f**,**f′**): N21.

The SEM images of the surfaces and cross-sections of the NIPS membranes are shown in Figure 4. The surface images reveal that the addition of GNPs to the system does not cause any heterogeneities. Also, it does not alter the uniform and smooth structure of the membranes, indirectly indicating proper dispersion of GNPs in the polymer matrix (no significant GNP aggregation) [33,49,50]. The cross-sectional images reveal a dense, selective layer on the membrane surface, under which is a sublayer with finger-like pores is formed. The neat PEI membrane has a thicker top surface compared to the other membranes. Adding GNPs to the membrane decreases the thickness of the skin layer on the membrane surface, resulting in an increased PWF. The addition of Pluronic to PEI creates small pores at the top surface, as well as larger finger-like channels in the sublayer, leading to increased PWF. Additionally, in the presence of GNPs and Pluronic P-123, the finger-like channels are better connected, resulting in the formation of larger macrovoids, facilitating easier water passage through the membrane [51]. It is commonly understood that the polymer-poor phase created during phase inversion develops into the membrane pores [52]. Two key factors affect the membrane morphology: (1) the addition of hydrophilic additives to the dope solution accelerates the solvent–nonsolvent exchange, resulting in a finger-like substructure with rough surface and (2) the presence of additives increases the effective viscosity of the casting solution, which decelerates the solvent–nonsolvent separation, leading to a sponge-like substructure and a smoother top surface [51].

**Figure 5:**
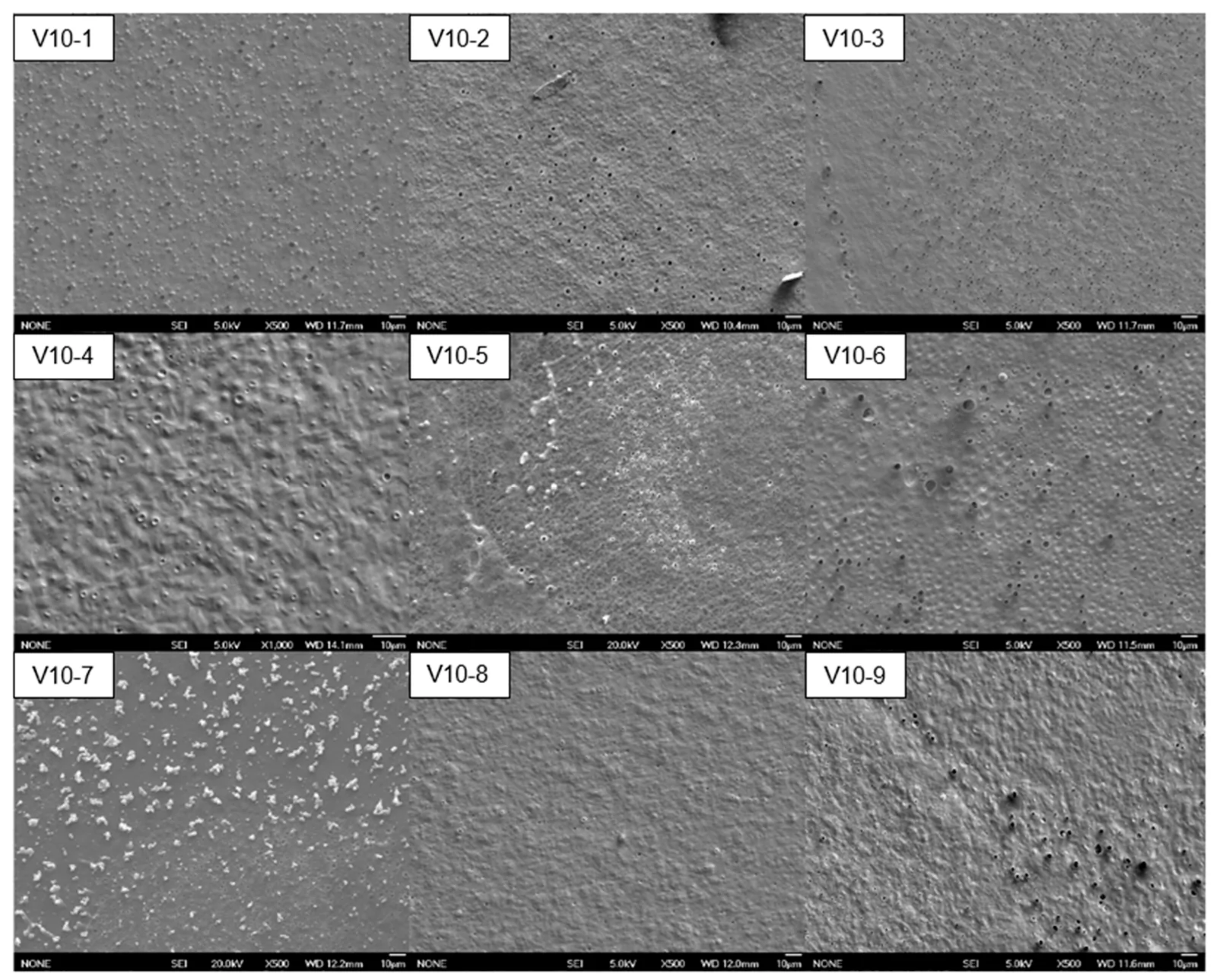
SEM images of the surfaces of the VIPS membranes.

**Figure 6:**
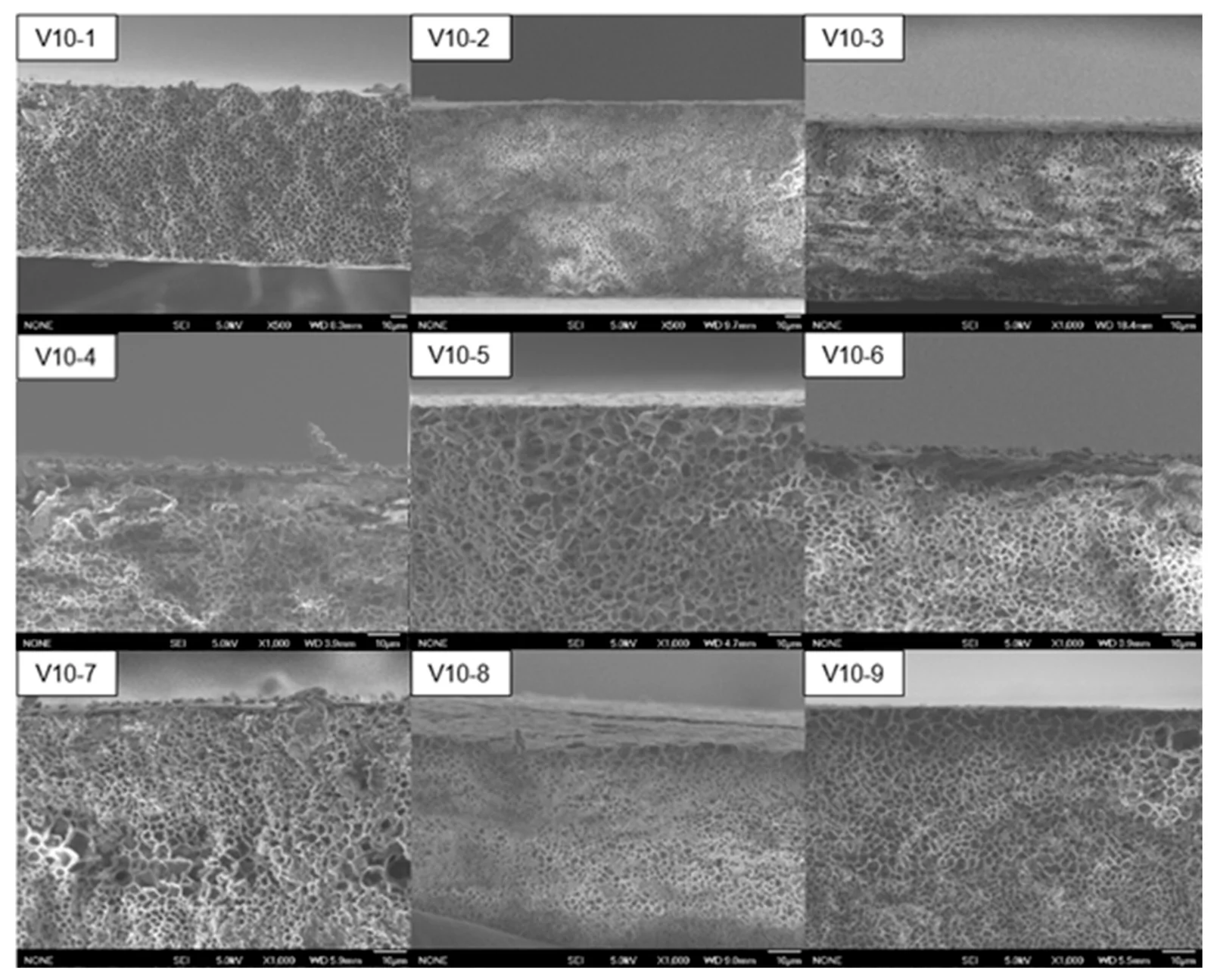
SEM images of the cross-sections of the VIPS membranes.

The SEM images of the surfaces and cross-sections of the VIPS membranes are shown in Figure 5 and Figure 6. These images and the related performance data offer valuable insights into how vapor exposure time and RH affect the structures and functions of the PEI membranes made using the VIPS method. At a fixed RH of 40%, increasing the vapor exposure time from 15 to 60 min changes the cross-sectional morphology from a relatively open cellular structure to a more compact and layered porous structure. This trend is observed in V10-1, V10-2, and V10-3. The V10-1 membrane shows a more uniformly distributed cellular pore network, whereas V10-2 and especially V10-3 exhibit a denser upper region and a more compact substructure. This suggests that longer vapor exposure times lead to greater polymer-rich phase consolidation before immersion in the coagulation bath, which reduces the effective pore volume and water-transport pathways. This interpretation is consistent with the porosity results, which decrease from 42% for V10-1 to 36% for V10-2 and 14% for V10-3. Regarding the effect of RH, comparison of the SEM images in Figure 5 and Figure 6 indicates that, under the investigated conditions, membranes fabricated at 40% RH generally exhibit a denser and more compact cellular structure than the corresponding membranes fabricated at higher RH levels. As RH rises to 65% and then to 90%, the membrane structure becomes more asymmetrical with larger pores. At 65% RH, the surfaces become rougher and the cross-sections show more developed cellular pores compared with the corresponding membranes prepared at 40% RH. Among these samples, V10-5 exhibits a relatively uniform cellular structure across the membrane thickness, suggesting a balanced rate of water vapor absorption and polymer precipitation. This morphology is consistent with its intermediate permeability and improved rejection compared with more open structures. This increase in the pore size with higher RH is linked to a rise in PWF, indicating better water permeability. The change from a symmetrical to an asymmetrical structure with higher RH shows improved pore formation due to increased water vapor absorption. At 90% RH, rapid water vapor absorption produces a more open and heterogeneous morphology. V10-7 shows a rougher surface and a coarse cellular cross-section, which is consistent with its higher PWF. However, with longer exposure at 90% RH, as observed for V10-8 and V10-9, the morphology becomes less uniform and partially compacted, indicating that prolonged vapor exposure allows pore coarsening and polymer-rich phase rearrangement before final immersion. In general, higher RH accelerates nonsolvent vapor uptake into the cast film, promoting earlier phase separation and the formation of a more open porous structure. This favors water permeability, especially at shorter exposure times. However, BSA rejection decreases (discussed later) with higher RH and longer vapor exposure times, suggesting that while higher RH improves water flux, it may reduce the membrane selectivity and separation performance. The different membrane structures are influenced by a balance between RH and vapor exposure time. Higher RH promotes pore development and connectivity, leading to higher PWF and potentially more open structures. On the other hand, longer vapor exposure times create denser and less permeable structures, decreasing PWF. Thus, the hydraulic performance is controlled not only by the apparent pore size, but also by the continuity of the skin region, pore connectivity, total porosity, and transport-path tortuosity. The changes in morphology are driven by both thermodynamic and kinetic factors, such as the rate of nonsolvent diffusion and mass transfer. Our findings can be interpreted through the fundamental mechanisms of phase inversion. In vapor-induced phase separation, RH controls the rate of water vapor penetration, while exposure time controls the extent of demixing and partial solidification before immersion in the coagulation bath. Phase separation mechanism plays a crucial role: a nucleation-and-growth demixing typically produces isolated, cell-like pores (macrovoids), whereas spinodal decomposition leads to bi-continuous porous networks [28]. The NIPS process in our study likely invokes instantaneous nucleation-driven demixing at the membrane surface, evident from the dense skin and finger-like voids in the neat PEI membrane (Figure 4) which is a morphology characteristic of fast solvent–nonsolvent exchange. In contrast, the VIPS membranes do not show the same finger-like substructure observed in the NIPS membranes. Instead, they exhibit cellular and compact porous morphologies whose development depends strongly on RH and vapor exposure time. Moreover, the variations observed within our VIPS-fabricated membranes are consistent with different extents of vapor-induced phase separation before immersion in the coagulation bath. A short vapor exposure (15 min) limits the time available for complete water vapor penetration and polymer precipitation throughout the cast film. Therefore, the subsequent immersion step can still contribute to additional nonsolvent-induced demixing, producing a more open cross-sectional morphology, particularly at higher RH. With longer exposure (30 and 60 min), the entire film has more time to absorb water vapor and undergo partial phase separation before immersion. As a result, the polymer-rich phase becomes more consolidated, and the final immersion step produces less abrupt pore formation. This leads to a denser effective transport structure, lower porosity, and reduced PWF. This mechanistic understanding explains the trade-off we observed, i.e., longer vapor exposure generally produced higher BSA rejection due to a tighter overall pore network but lower pure water flux, whereas shorter exposure and higher RH favored a more open pore structure, increasing water flux but lowering rejection. In essence, VIPS enables morphology tuning through the competition between vapor-induced pore formation and polymer-rich phase consolidation before final coagulation. These observations are qualitatively consistent with AlMarzooqi et al. [53], who also reported distinct membrane morphologies resulting from NIPS and VIPS and related these differences to the different rates and mechanisms of nonsolvent uptake during phase separation. Their results further demonstrate that membrane transport cannot be interpreted solely from mean pore size, since structural characteristics such as pore connectivity, tortuosity, pore density, and surface morphology also contribute to transport resistance. Although their study involved PVDF membranes for membrane distillation, the reported NIPS–VIPS structure–formation relationships provide useful mechanistic support for the trends observed in the present PEI ultrafiltration membranes.

#### 3.1.5. Membrane Mechanical Properties

**Figure 7:**
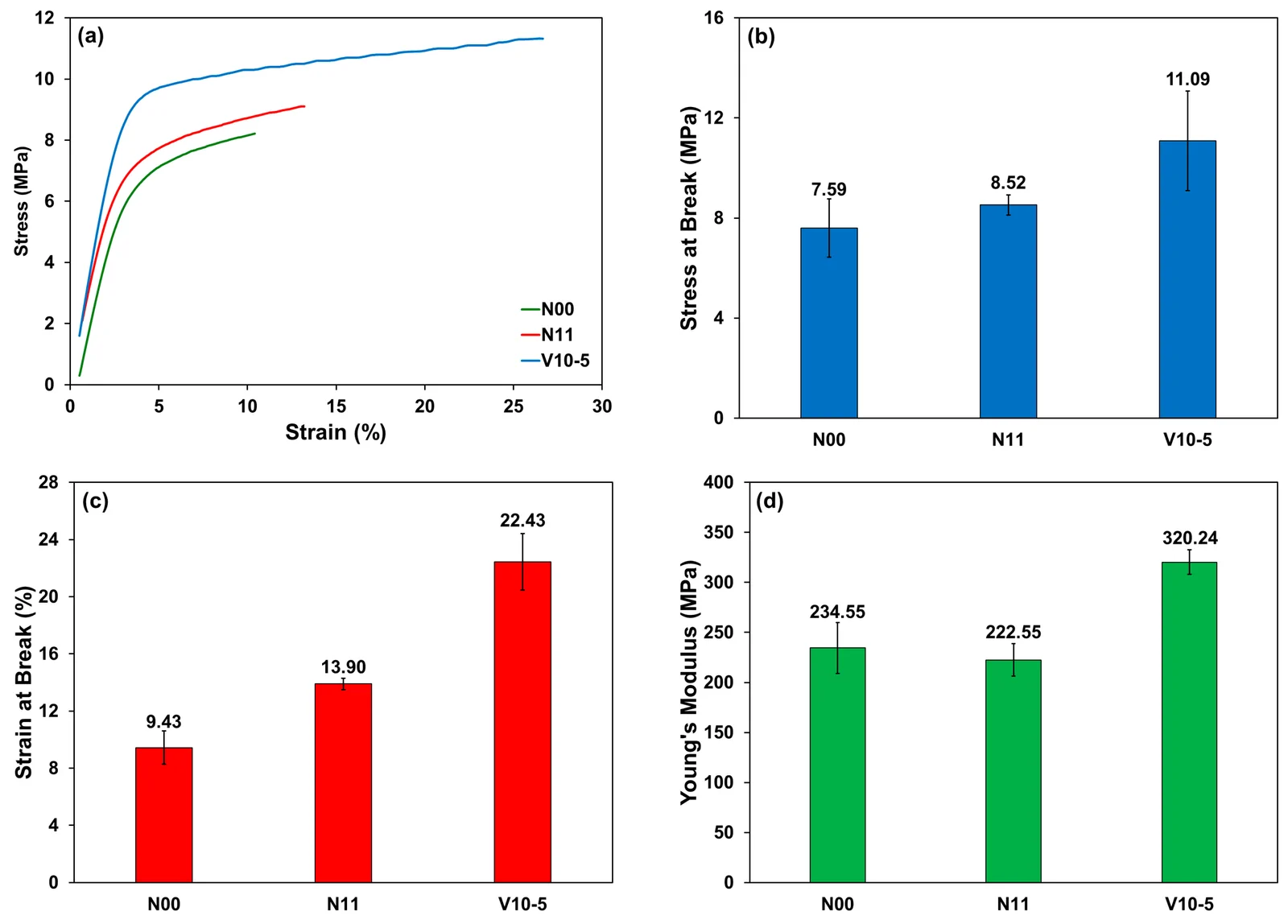
Quasi-static tensile properties of the NIPS (Samples N00 and N11) and VIPS membranes (Sample V10-5): (**a**) stress–strain curves, (**b**) stress at break, (**c**) strain at break, (**d**) Young’s modulus.

The quasi-static tensile properties of select NIPS and VIPS membranes are shown in Figure 7a. In general, GNPs are known to significantly improve the mechanical properties of polymer/elastomer composites [54]. However, herein, the membranes contain both GNPs and Pluronic P-123, necessitating the consideration of their combined effect on the mechanical properties of the membranes. Notably, Pluronic P-123 at concentrations as low as 0.2 wt.% is observed to significantly reduce the mechanical properties of membranes [55]. This behavior is attributed to the interaction between the hydrophobic PPO blocks in Pluronic P-123 and the PEI macromolecules. Furthermore, even small amounts of Pluronic P-123 can induce macrovoid formation [56]. In Figure 7b, the stress at break increases with the addition of GNPs and Pluronic P-123, as evidenced in Sample N11. It is important to note that the increase in stress at the break for the VIPS membrane sample (V10-5) is due to the asymmetric cellular morphology chain which exhibited greater overall flexibility during testing. Meanwhile, with respect to the strain at break for the samples (Figure 7c), it is observed that Pluronic P-123 (Sample N11) mitigates the rigidity imposed by graphene layers, leading to a higher strain at break value than that of Sample N00. However, the V10-5 sample demonstrates that morphological changes can further increase strain at break. Regarding Young’s modulus (Figure 7d), the V10-5 sample again shows the best results, consistent with literature reports on an observed increase in elongation at break at specific exposure times [57]. However, in the Hookean region, sample N11 proves that Pluronic P-123 has a more pronounced effect on Young’s modulus than GNPs.

### 3.2. Membrane Performance

#### 3.2.1. PWF and BSA Rejection of the NIPS Membranes

**Figure 8:**
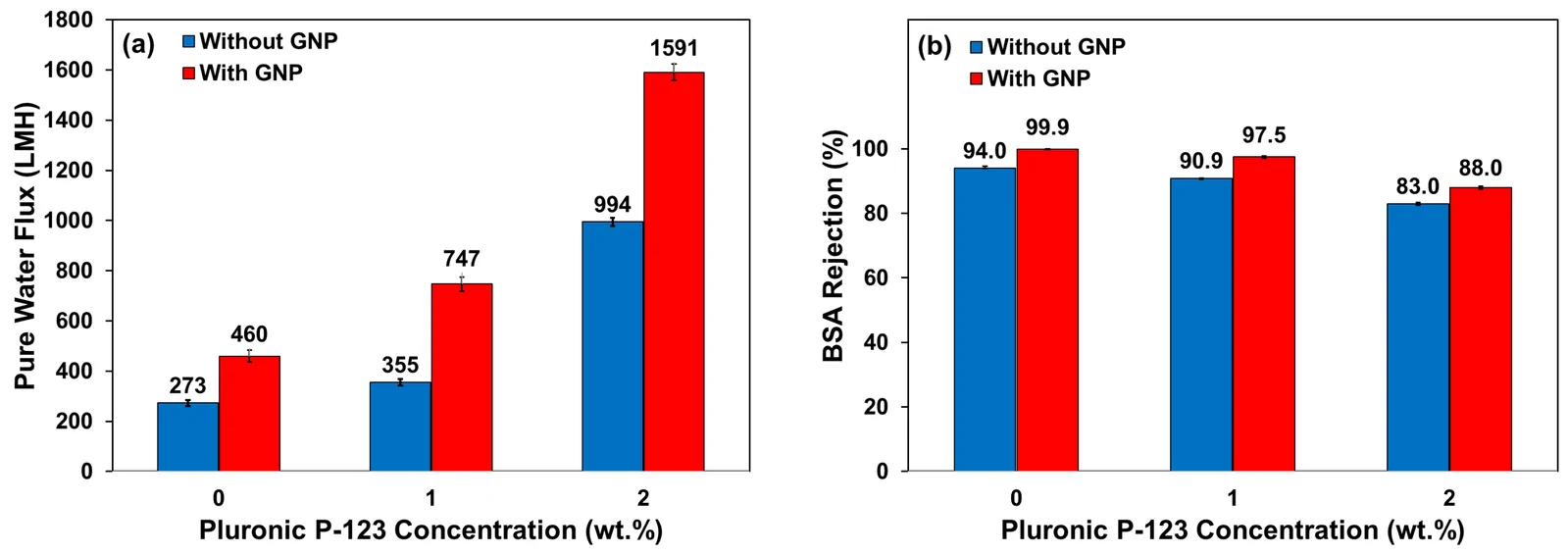
(**a**) Pure water fluxes and (**b**) BSA rejection of the membranes fabricated using the NIPS method.

In Figure 8a, the PWF of the membranes fabricated using the NIPS method are shown, where the effects of varying the concentration of Pluronic P-123 and adding GNPs to the membranes can be elucidated. Based on this figure, increasing the amount of Pluronic P-123 from 0 to 2 wt.% leads to a significant increase in PWF. For example, the PWF of the neat membrane (N00) is 272 LMH, while it reaches 994 LMH for N20, indicating a 3.65-fold enhancement in PWF. This illustrates the role of Pluronic P-123 as a porogen, which increases the porosity and pore sizes of the membranes during the phase inversion process. Additionally, the incorporation of 1 wt.% GNPs into each sample, regardless of the Pluronic P-123 content, further increases the PWF due to thinning of the selective layer without loss of polymer morphology or porosity resulting from Pluronic P-123. It is noted these changes are evidenced by qualitative interpretation of SEM images correlated with PWF experiments. Thus, the optimal PWF among all NIPS samples belongs to N21 with a value of 1591 LMH. This encompasses the effect of both Pluronic P-123 and GNPs.

In Figure 8b, the BSA rejection of the membranes fabricated using the NIPS method are provided. In this figure, the effects of varying the concentration of Pluronic P-123 and adding GNPs to the membrane can be elucidated. As expected, increasing the concentration of Pluronic P-123 leads to a decrease in the BSA rejection of the membranes due to increased porosity and pore size. However, the addition of GNPs enhances rejection not only by increasing hydrophilicity, but also by modifying the membrane morphology and reducing nonselective transport pathways through the selective skin layer. In asymmetric UF membranes, BSA rejection is governed mainly by the effective pore constrictions in the dense surface layer, pore-size distribution, pore connectivity, and possible protein–membrane interactions, rather than by the apparent mean hydraulic pore size calculated from water flux alone. In general, although N20 and N21 show the highest PWF results, their rejection drops below 90%, which is not desirable. Specifically, N20 has a rejection of 83%, while the presence of GNPs in N21 improves the rejection to 88%. Conversely, the neat membranes with and without GNPs show the highest rejection, with N00 achieving 94% and N01 reaching almost 100%. However, their PWF values are too low to be acceptable. The membrane containing 1 wt.% Pluronic P-123, i.e., N10, has a PWF of 355 LMH and a rejection of 91%. The presence of GNPs in N11 significantly increases both the PWF and the rejection to 747 LMH and 98%, respectively. Therefore, N10 and N11 may be selected as the optimal NIPS membranes, showing the best performance with and without GNPs. The high rejection of N10 and N11, despite their relatively large apparent hydraulic pore sizes, can therefore be explained by the presence of a dense selective skin layer that controls BSA transport, while the underlying finger-like porous substructure mainly supports high permeability. This structure–performance relationship is consistent with the SEM observations of the NIPS membranes. These membranes were selected for further investigation.

#### 3.2.2. PWF and BSA Rejection of the VIPS Membranes

**Figure 9:**
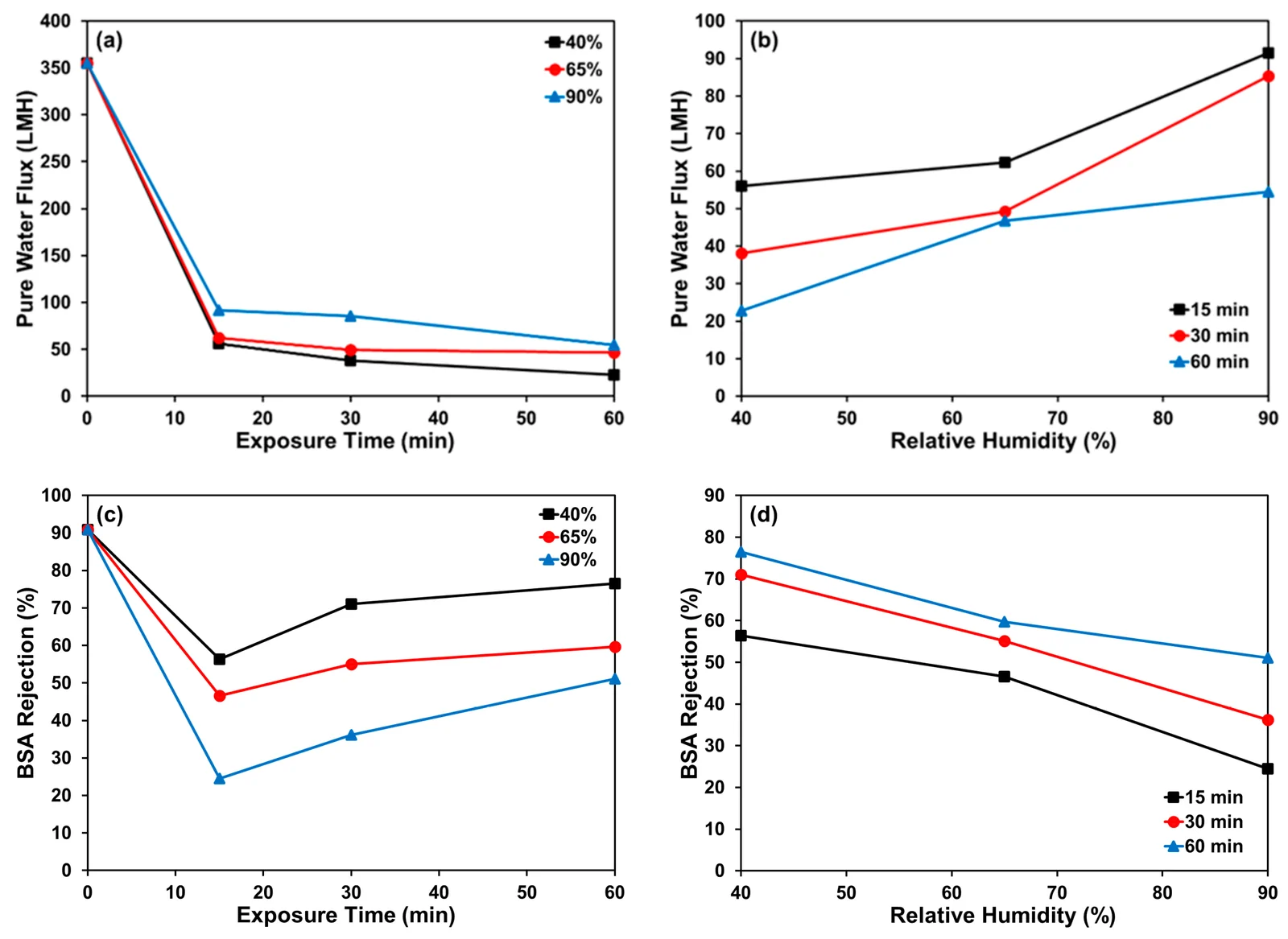
Pure water fluxes of the membranes fabricated using the VIPS method as a function of (**a**) vapor exposure time at different relative humidity (40, 65, and 90%) and (**b**) relative humidity at different vapor exposure time (15, 30, and 60 min); the bovine serum albumin (BSA) rejection of the membranes fabricated using the VIPS method as a function of (**c**) vapor exposure time and (**d**) relative humidity. Data are presented as the mean ± standard deviation (n=3). In all panels, the data at zero vapor exposure time correspond to the composition-matched N10 membrane fabricated using NIPS.

Because all V10-x membranes contain 1 wt.% Pluronic P-123 and no GNPs, the effect of the fabrication pathway was evaluated exclusively by comparison with N10. The GNP-containing N11 and N21 membranes were not used for this fabrication-route comparison and are discussed separately in Section 3.2.1 as part of the evaluation of GNP incorporation. The PWF for membranes fabricated using the VIPS method are shown in Figure 9a,b. A vapor exposure time of zero in this figure represents the NIPS method so that a comparison can be made between the NIPS and VIPS methods with respect to the membrane performance at constant Pluronic P-123 concentration. As seen in Figure 9a, changing the fabrication method from NIPS to VIPS significantly decreases the PWF of the membranes. This observation represents a change in the morphology of the VIPS membranes, which aligns with the morphology studies discussed before. The results also demonstrate that both vapor exposure time and RH significantly influence the PWF of the PEI membranes containing Pluronic P-123 and fabricated using the VIPS method. As vapor exposure time increases, PWF decreases for all RH levels, with the sharpest decline observed at 40% RH. At 90% RH, the decrease in PWF is more gradual, suggesting that higher humidity levels mitigate the densification process of the membrane (Figure 9a). This trend aligns with porosity calculation in previous sections. RH plays a critical role in maintaining membrane porosity. Higher RH levels result in higher PWF, regardless of the vapor exposure time. For example, at 15 min, PWF increases from 56 LMH at 40% RH to 91 LMH at 90% RH (Figure 9b). This positive correlation between RH and PWF is consistent with the observations of porosity and apparent mean hydraulic pore size, as explained before. The interplay between vapor exposure time and RH suggests that optimizing these parameters is crucial for tailoring membrane properties. For short vapor exposure times, higher RH significantly boosts PWF, while longer vapor exposure times lead to reduced flux, particularly at lower humidity. This balance is crucial for applications requiring specific membrane characteristics.

In Figure 9c,d, the BSA rejection for the membranes fabricated using the VIPS method are provided. A vapor exposure time of zero in this figure represents the membrane fabricated using the NIPS method so that a comparison can be made between the performances of the membranes fabricated using the NIPS and VIPS methods at a constant Pluronic P-123 concentration. As observed in Figure 9c, changing the fabrication method from NIPS to VIPS significantly decreases the BSA rejection performances of the membranes. This observation represents a change in the morphology of the VIPS membranes, which aligns with the morphology studies discussed before. For example, the BSA rejection decreases from ~91% for the NIPS membrane to 56% for the VIPS membrane at 40% RH and after a vapor exposure time of 15 min, followed by an increase to 76.5% BSA rejection at the same relative humidity and after 60 min vapor exposure time (Figure 9c). The initial decrease can be attributed to significant morphological changes occurring when switching from NIPS to VIPS. In particular, non-finger-like structures are observed that are less dense but more uniform, thereby negatively impacting both PWF and rejection. However, as vapor exposure time increases beyond 15 min, the rejection efficiency improves, indicating a gradual formation of a denser and more selective membrane structure, which aligns with the observed decrease in PWF as discussed previously. This trend is consistent with the findings of Barambu et al. [58] who noted similar improvements in the rejection of oil in an oil/water mixture filtered using a PSF membrane, when it was fabricated using the VIPS method at longer vapor exposure times under controlled humidity conditions.

When examining the effect of RH on membranes at specific vapor exposure times, increasing the RH generally leads to a decrease in the BSA rejection. At 15 min exposure, the BSA rejection drops from 56% at 40% RH to 24.5% at 90% RH (Figure 9d). This trend continues at longer vapor exposure times (30 and 60 min), where higher RH levels lead to reduced BSA rejection efficiency, corroborating the observation that higher RH results in more open membrane structures. Overall, the interplay between vapor exposure time and RH is crucial for optimizing membrane performance. Lower RH and increased vapor exposure times tend to produce membranes with higher BSA rejection efficiency, while higher RH levels do the opposite. These results are consistent with the broader literature on membrane fabrication via VIPS, reiterating the importance of controlling the phase separation conditions to achieve desired membrane properties.

Two-way ANOVA confirmed that both VIPS processing parameters significantly influenced membrane performance. For PWF, significant effects were observed for RH [F,(2,18)=830.06 p<0.0001], vapor exposure time [F,(2,18)=456.59 p<0.0001], and their interaction [F,(4,18)=45.66 p<0.0001]. Similarly, BSA rejection was significantly affected by RH [F,(2,18)=724.95 p<0.0001], vapor exposure time [F,(2,18)=307.56 p<0.0001], and the RH–exposure time interaction [F,(4,18)=14.97 p<0.0001]. The significant interaction terms indicate that the influence of vapor exposure time depended on the applied RH. Therefore, the effects of RH and exposure time should be interpreted jointly, consistent with the response profiles shown in Figure 9.

The direct comparison between N10 and the V10-x series demonstrates the effect of the fabrication pathway independently of GNP incorporation. N10 exhibited a PWF of 355 LMH and approximately 91% BSA rejection. In comparison, the highest PWF among the VIPS membranes was 91 LMH for V10-7, while the highest BSA rejection was 76.5% for V10-3. The selected balanced VIPS condition, V10-5, exhibited a PWF of 49 LMH and a BSA rejection of 55%. Therefore, under the investigated processing conditions, the composition-matched NIPS membrane outperformed the VIPS membranes in both permeability and selectivity. The higher performance of N11 and N21 is discussed separately as an effect of GNP incorporation and does not form the basis of the NIPS–VIPS comparison. The SEM images of the surfaces and cross-sections of the membranes fabricated using the NIPS and VIPS methods (Figure 4, Figure 5 and Figure 6) reveal that the NIPS membranes feature a more distinct dense selective skin layer supported by a porous finger-like substructure. This asymmetric structure explains the superior BSA separation performance of the NIPS membranes: the dense surface layer provides the main resistance to BSA passage, while the porous sublayer reduces hydraulic resistance and supports higher water flux. In contrast, the VIPS membranes do not exhibit a selective skin layer as pronounced as that of the NIPS membranes, because their porous structure extends closer to the top surface. Consequently, their BSA rejection is lower despite their lower apparent hydraulic pore sizes. This comparison confirms that BSA rejection is controlled primarily by the effective pore constrictions and continuity of the selective layer, rather than by the apparent mean hydraulic pore size alone. To further contextualize the permeability–selectivity performance of the optimized membranes, Table 5 compares the present results with representative polymeric ultrafiltration membranes reported in the literature.

As summarized in Table 5, N11 exhibits a favorable combination of PWF and BSA rejection relative to several previously reported polymeric UF membranes. Within the present PEI system, incorporation of 1 wt.% GNPs increased the PWF from 355 LMH for N10 to 747 LMH for N11 while simultaneously increasing BSA rejection from approximately 91% to 98%. However, direct comparison of absolute PWF values among different studies should be made cautiously because operating pressure, membrane composition, membrane thickness, feed conditions, and filtration configuration vary considerably. Several reported membranes also exhibit comparable or higher permeability–selectivity performance under different operating conditions. Therefore, the literature comparison supports the favorable permeability–selectivity balance of N11 within the present system rather than establishing universal superiority over previously reported UF membranes.

#### 3.2.3. Antifouling Properties

Fouling can cause serious deficiencies in membrane performance, such as reduced water flux, decreased membrane durability, and increased maintenance cost [70,71]. Two types of fouling can occur during membrane operation: (1) reversible fouling and (2) irreversible fouling. Reversible fouling, which can be alleviated by cleaning the membrane surface, is caused by weak adsorption of BSA onto the membrane surface. Irreversible fouling, which affects membrane lifespan, is caused by strong adsorption of BSA on the membrane surface or entrapment of BSA within the membrane pores [33]. The presence of hydrophilic nanomaterials such as GNPs can significantly enhance the antifouling properties of the UF membranes [72]. The membrane antifouling behavior is strongly influenced by the hydrophilicity of the membranes, as higher hydrophilicity translates to more resistance to fouling. In Table 6, the antifouling parameters of the NIPS membranes and the optimal VIPS membrane, V10-5, are shown. The antifouling properties of membranes modified with GNPs are improved compared to similar membranes without GNPs, a trend also observed with the addition of Pluronic P-123. The FRR increases by increasing the GNP and Pluronic P-123 contents up to 1 wt.%. This enhancement is attributed to the increased hydrophilicity of the membranes, consistent with the WCA measurements of the fabricated membranes. The hydrophilic functional groups on the membrane surface carry a negative charge, which leads to significant electrostatic repulsion against the negatively charged BSA molecules at neutral pH [33]. However, further increasing the Pluronic P-123 content results in a reduction in FRR. This can be attributed to the strong BSA absorption caused by the increase in membrane pore size. The BSA particles can penetrate the membrane sublayer and restrict overall permeation through the membrane. Thus, the irreversible fouling ($R_{ir}$) increases and FRR decreases. The antifouling properties of V10-5 are significantly weaker than those of the NIPS membranes, which typically have a dense top layer with smaller pore sizes and smoother surfaces, as discussed in Section 3.1.4. The NIPS surfaces act as barriers to the foulant attachment, thereby enhancing the antifouling properties of the membranes. Furthermore, the lower hydrophilicity of the VIPS membrane reduces their fouling resistance. The more heterogeneous pore size distributions of the VIPS membranes also allow deeper foulant penetration, making membrane cleaning and regeneration more challenging. Thus, an increase in $R_{ir}$ and a reduction in FRR is observed.

## 4. Conclusions

Polyetherimide (PEI) membranes with 0, 1, and 2 wt.% Pluronic P-123, with and without graphene nanoplatelets (GNPs) (0–1 wt.%), were fabricated and evaluated using the nonsolvent-induced phase separation (NIPS) method. The results showed that the membrane with 1 wt.% Pluronic P-123 had promising bovine serum albumin (BSA) separation performance, with a pure water flux (PWF) of 355 LMH and a rejection of ~91%. Adding 1 wt.% GNPs to this membrane further improved its separation performance, yielding a PWF of 747 LMH and a rejection of 98%. This N10–N11 comparison represents the effect of GNP incorporation within the NIPS series and was not used to assess the effect of the fabrication pathway. The main advantage of this optimized NIPS membrane is its favorable permeability–selectivity balance, achieved through an asymmetric structure in which the dense selective skin layer governs BSA rejection while the porous substructure supports high water permeability. The neat PEI membrane had a PWF of 272 LMH and a rejection of 94%. Increasing the Pluronic P-123 content to 2 wt.% increased the PWF to 994 LMH in the absence of GNPs and 1591 LMH in the presence of GNPs, but reduced the rejection to 83% and 88%, respectively. The antifouling properties of the membrane also declined with higher Pluronic P-123 content due to increased pore size, showing a flux recovery ratio (FRR) of 73.5% and a total reflux ratio (Rt) of 45.2% for the 1 wt.% Pluronic P-123 membrane. With GNP addition, FRR increased to 78.5% and Rt decreased to 38%. This was a significant improvement compared to the neat PEI membrane, which had an FRR of 64.2% and Rt of 50.5%. The hydrophilicity study indicated that adding Pluronic P-123 and GNPs increased the membrane hydrophilicity, with water contact angle (WCA) measurements of 60.9° and 56.4° for membranes containing 1 wt.% Pluronic P-123 without and with GNPs, respectively, compared to 67.5° for the neat membrane.

The effect of the fabrication pathway was evaluated separately by comparing N10 with the composition-matched V10-x membranes, all of which contained 1 wt.% Pluronic P-123 and no GNPs. Longer vapor exposure times generally decreased PWF but increased BSA rejection, whereas higher RH increased PWF but decreased rejection. N10 exhibited a PWF of 355 LMH and approximately 91% BSA rejection. In comparison, the highest PWF among the VIPS membranes was 91 LMH, and the highest BSA rejection was 76.5%. The selected balanced VIPS membrane, V10-5, fabricated at 65% RH for 30 min, exhibited a PWF of 49 LMH and BSA rejection of 55%. Therefore, the conclusion that NIPS outperformed VIPS is based specifically on the composition-matched comparison between N10 and the V10-x series, rather than on comparisons involving the GNP-containing N11 or N21 membranes. This comparison demonstrates that the fabrication route controls not only membrane porosity and apparent hydraulic pore size, but also the continuity of the selective skin layer, which is critical for BSA rejection. The antifouling properties of this VIPS membrane showed an FRR of 18.5% and Rt of 94%, which were not acceptable compared to the NIPS membrane. It seems that the separation mechanism in this system is more size selective rather than affinity-based separation. The results demonstrate that PEI membranes with 1 wt.% Pluronic P-123 and 1 wt.% GNPs fabricated using the NIPS method have acceptable and promising BSA separation performance. It is noted that additional studies can demonstrate statistical significance and validate the observed performance trends. Furthermore, additional studies characterizing molecular-weight cutoff and rejection performance with other model proteins may provide further insight on the influence of Pluronic-123 and GNPs on PEI UF membrane performance.

## Figures and Tables

**Table 1:** Casting solution compositions.

Membrane ^a^	Fabrication Method	PEI (wt.%)	Pluronic P-123 (wt.%)	GNPs (wt.%)
**N00**	NIPS	17	0	0
**N01**	NIPS	17	0	1
**N10**	NIPS	17	1	0
**N11**	NIPS	17	1	1
**N20**	NIPS	17	2	0
**N21**	NIPS	17	2	1
**V10-1 to V10-9**	VIPS	17	1	0

^a^ In the membrane designations, N and V refer to the NIPS and VIPS methods, respectively. Also, the first and second numeric values refer to the percentages of Pluronic P-123 and GNPs, respectively.

**Table 2:** Process parameters for the membranes fabricated using VIPS at 50 °C.

Membrane	Relative Humidity (RH) (%)	Vapor Exposure Time (min)
**V10-1**	40	15
**V10-2**	40	30
**V10-3**	40	60
**V10-4**	65	15
**V10-5**	65	30
**V10-6**	65	60
**V10-7**	90	15
**V10-8**	90	30
**V10-9**	90	60

**Table 3:** Porosities and apparent mean hydraulic pore size of the NIPS membranes.

Membrane	Porosity (%)	Apparent Mean Hydraulic Pore Radius (nm)
**N00**	32	26
**N01**	34	32
**N10**	35	28
**N11**	37	39
**N20**	38	44
**N21**	38	55

**Table 4:** Porosity and apparent mean hydraulic pore size of the VIPS membranes.

Membrane	Porosity (%)	Apparent Mean Hydraulic Pore Radius (nm)
**V10-1**	42	9.8
**V10-2**	36	8.9
**V10-3**	14	11.9
**V10-4**	48	9.4
**V10-5**	29	11.6
**V10-6**	16	15.9
**V10-7**	46	11.7
**V10-8**	41	12.2
**V10-9**	29	12.2

**Table 5:** Comparison of the permeability–selectivity performance of the selected membranes in this study with representative polymeric ultrafiltration membranes reported in the literature.

Base Polymer	Additive	Fabrication Method	Operating Pressure (MPa)	Protein	PWF (LMH)	Rejection (%)	Ref.
**PEI**	Pluronic P-123	NIPS	0.6	BSA	355	91	This study
**PEI**	Pluronic P-123 + GNP	NIPS	0.6	BSA	747	98	This study
**PEI**	Pluronic P-123	VIPS	0.6	BSA	49	55	This study
**PEI**	PVP	NIPS	0.345	BSA	147.1	85.2	[45]
**PEI**	Pluronic F-127	NIPS	0.345	BSA	255	84	[59]
**PEI**	Exfoliated MoS_2_	NIPS	0.414	BSA	52.54	94.5	[60]
**PPSU**	PVP + GO	NIPS	0.2	BSA/pepsin	171	95	[61]
**PES**	ITF	NIPS	0.1	BSA	319.4	95.9	[62]
**PES**	PSFNA	NIPS	0.1	BSA	478	97.5	[63]
**PES/SPSF**	GO	NIPS	0.1	BSA	816.9	>99.2	[64]
**PES**	MoS_2_-PDA-PEI	NIPS	0.1	BSA	364.03	96.5	[65]
**PPESK**	PEG400 + LiCl	NIPS	0.1	γ-globulin	1139	93.7	[66]
**PES**	Tetrabutylammonium chloride/imidazole DES	NIPS	0.2	BSA	781	97.7	[67]
**PES/SPSF**	SPSF	RTIPS	0.1	BSA	966	79.2	[68]
**PMIA**	PEG400 + LiCl	NIPS	0.1	BSA	239.63	98.2	[69]

**Note:** PWF values were obtained under different experimental conditions and were not normalized by transmembrane pressure. Differences in operating pressure, membrane composition and thickness, feed concentration, filtration configuration, and testing procedure should therefore be considered when comparing absolute flux values. The comparison is intended to contextualize the permeability–selectivity balance rather than provide a direct performance ranking. Commercial-membrane performance was not reported due to insufficient data present in the literature.

**Table 6:** Antifouling properties of the fabricated membranes.

Membrane	*FRR* ^a^ (%)	*R_t_* ^b^ (%)	*R_ir_* ^c^ (%)	*R_r_* ^d^ (%)
**N00**	64.2	50.5	35.8	14.7
**N01**	72.7	44.8	27.3	17.5
**N10**	73.5	45.2	26.6	18.6
**N11**	78.5	38.0	21.5	16.5
**N20**	58.4	50.9	41.6	9.3
**N21**	51.4	59.1	48.6	10.4
**V10-5**	18.5	94.0	81.5	12.5

^a^ flux recovery ratio; ^b^ total fouling ratio; ^c^ irreversible fouling ratio; ^d^ reversible fouling ratio.

## Data Availability

The data presented in this study are available on request from the corresponding authors.
